# Coordinated repressive chromatin-remodeling of *Oct4* and *Nanog* genes in RA-induced differentiation of embryonic stem cells involves RIP140

**DOI:** 10.1093/nar/gku092

**Published:** 2014-01-30

**Authors:** Cheng-Ying Wu, Xudong Feng, Li-Na Wei

**Affiliations:** Department of Pharmacology, University of Minnesota Medical School, Minneapolis, MN 55455, USA

## Abstract

Maintaining pluripotency and indefinite self-renewal of embryonic stem cells requires a tight control of the expression of several key stemness factors, particularly Nanog and Oct4 transcription factors. The mammalian SWItch/Sucrose NonFermentable (SWI/SNF) complex contains Brg1 or Brm as its core subunit, along with Brg1-associated factors. Our previous studies have addressed chromatin-remodeling of the *Oct4* gene locus in retinoic acid (RA)-treated embryonal carcinoma cell line P19, which involves receptor-interacting protein 140 (RIP140) for heterochromatinization on the proximal promoter region of this gene locus. However, the mechanism of RIP140 action in RA-triggered repressive chromatin-remodeling is unclear. The current study examines RA repression of the *Nanog* gene and compares the results with RA repression of the *Oct4* gene on the chromatin level. The results show a loose nucleosome array on the *Nanog* gene promoter in undifferentiated embryonic stem cells. On RA treatment, the *Nanog* gene locus remodels specifically in the CR1 region of its proximal promoter, with the insertion of a nucleosome and compaction of this region. Further, RA induces coordinated chromatin-remodeling of both *Nanog* and *Oct4* gene loci, which requires RA receptor-α, RIP140 and Brm. Finally, in these RA-triggered repressive chromatin-remodeling processes, lysine acetylation of RIP140 is critical for its recruiting Brm.

## INTRODUCTION

Controlling the expression levels of Nanog and Oct4 transcription factors as well as other stemness factors is required for maintaining pluripotency and indefinite self-renewal of embryonic stem cells (ESCs) (1–3). Nanog and Oct4 are particularly crucial for ESCs to stay in their ground states (2). For instance, ectopic over-expression of Nanog and Oct4 drives cytokine-independent self-renewal in undifferentiated ESCs and prevents ectoderm differentiation (2). Gene knockout of Nanog or Oct4 affects early embryonic development (4,5). Studies have also shown that precise levels of Nanog and Oct4 are needed for ESCs to acquire their ground states and for maintaining their pluripotency (3,6). We previously examined the chromatin conformation of the *Oct4* gene promoter and its chromatin-remodeling in retinoic acid (RA)-induced differentiation of embryonal carcinoma P19 cells and reported heterochromatinization of the *Oct4* gene locus in RA-treated cells, which involved receptor-interacting protein 140 (RIP140) (7). This is consistent with the widely observed silencing of this gene locus in both differentiated ESC and P19 cells. However, whether the other stemness gene, *Nanog*, undergoes chromatin-remodeling in RA-differentiated stem cells is not clear. The mechanism underlying RIP140’s action is also not understood.

In ESCs, a specific esBrg1-associated factor (BAF) complex is required for maintaining stable expression of the stemness transcription factors Nanog, Oct4 and Sox2 (8,9). The SWI/SNF complex acts in chromatin-remodeling to maintain proper gene expression. In mammalian cells, either Brg1 or Brm serves as the central subunit along with a number of BAFs to form SWI/SNF complexes (9). Depending on the context, Brm can functionally compensate for Brg1 (10), compact chromatin during differentiation and associate with BAF155 to become the neural progenitor BAF complex (npBAF) complex in neuronal differentiation. During ESC differentiation, turning off the stemness genes is essential for differentiation programs to occur. The detail of how the BAF complex shields and silences these stemness gene loci in cell differentiation is unclear.

ESCs can differentiate into all three-cell lineages (i.e. endoderm, mesoderm and ectoderm). RA is one of the most widely used differentiation agents, whose action is mediated by two super families of retinoid receptors, retinoid X receptors and RA receptors (RAR) that form heterodimers in a ligand-dependent manner and bind to RA-responsive elements to regulate RA-target gene expression. Previously, our laboratory has reported ligand-dependent interaction of RIP140 with RAR and retinoid X receptors and identified RIP140 as a negative modulator of RA-regulated gene expression that was able to recruit several gene-repressive cofactors such as HDACs, CtBP and G9a (11–13). Additionally, we reported extensive post-translation modifications on RIP140, which could augment its gene regulatory activity (14–16). The physiological function of RIP140 includes its activity in regulating metabolism, reproduction, behavior and immunity, as shown in studying whole body and tissue-specific gene knockout or knockdown mice (17–19). In *in vitro* models, RIP140 has been shown to be involved in several cell differentiation systems such as adipocyte (13,20) and P19 stem cells (21,22); however, whether the RIP140 complex plays a role in modulating the stemness features of ESCs has not been addressed.

In the present study, we have uncovered a functional role for acetylated RIP140 in RA-triggered repressive chromatin-remodeling of both the *Nanog* and *Oct4* gene loci through complex formation with RAR-α and Brm, which replaces Brg1 on these gene promoters. The studies also define the chromatin conformation and nucleosome organization of the *Nanog* gene promoter in ground state ESCs and compare RA-triggered repressive chromatin-remodeling processes of *Nanog* and *Oct4* gene promoters. The results show that lysine-acetylated RIP140 is required for gene repression, which facilitates Brm exchange with Brg1 on *Nanog* and *Oct4* gene promoters; however, this phenomenon is not detected on other RA-repressive genes such as *Sox2.* These results establish an interesting RA-triggered, coordinated chromatin-remodeling process on *Nanog* and *Oct4* gene loci, which is dependent on acetylated RIP140 that facilitates the recruitment of Brm to replace Brg1 on these promoters.

## MATERIALS AND METHODS

### Cell culture and treatment

CJ7 and B16 ESCs were maintained in ES medium (Dulbecco’s modified Eagle’s medium), supplemented with 17% ESC-qualified fetal bovine serum, 2 mM glutamine, 0.1 mM nonessential amino acids, 6 μM β-mercaptoethanol, 2 mM HEPES and 1000 U/ml recombinant leukemia inhibitory factor. Cells were grown on irradiated mouse embryonic feeder cells in 0.2% gelatin-coated plates. For monoculture differentiation, undifferentiated ESCs were dissociated and plated onto 0.2% gelatin-coated tissue culture plastic at a density of 0.5–1.5 × 10^4^/cm^2^ in regular medium without leukemia inhibitory factor. Medium was renewed every day. For RA treatment, experiments were conducted using 1 μM RA for indicated time periods.

### RNA interference

RIP140-small interfering RNA (siRNA) and RAR-α-siRNA were purchased from Qiagen. Brm-siRNA and Brg1-siRNA were purchased from Sigma (St. Louis, MO, USA, www.sigmaaldrich.com/united-states.html). siRNAs were introduced into cells by HiPerFect transfection reagent (301704, Qiagen, Hilden, Germany, www1.qiagen. com). The cells were retransfected every 2 days and subcultured into a monolayer after the second transfection (i.e. on day 6).

### Reverse transcription and real-time polymerase chain reaction

Total RNA was extracted from EC cells using Trizol reagent according to the manufacturer’s instructions (Invitrogen). RNA was reverse transcribed by using the Omniscript RT kit (205113, Qiagen). For real-time polymerase chain reaction (PCR) analysis, 2× Brilliant II Master Mix (600804, Agilent Technologies, Santa Clara, CA, USA, www.home.agilent.com) was used, and PCR was performed on an MX3000P Stratagene thermocycler. The relative values were normalized to β-actin and presented as ΔΔCt methods (23). All the primers (for *Nanog*, *Oct4*, RIP140 and β-actin) were purchased from Qiagen.

### Chromatin immunoprecipitation assay

Chromatin immunoprecipitation (ChIP) assays were performed as described (20), using the following antibodies: BAF155 (sc-9746X, Santa Cruz Biotechnology), Brg1 (sc-10768X, Santa Cruz Biotechnology), Brm (sc-28710X, Santa Cruz Biotechnology), RIP140 (ab42126, Abcam, Cambridge, UK, www.abcam.com), H3K9me2 (ab1220, Abcam), H3K4me3 (07-473, Millipore), AcH3 (06-599, Millipore), HP1c (sc-10213, Santa Cruz Biotechnology), Oct4 *(*2750, cell signaling), Sox2 (sc-17320X, Santa Cruz Biotechnology), c-Jun (sc-1694, Santa Cruz Biotechnology), RAR-α (sc-551, Santa Cruz Biotechnology) and c-Fos (sc-48869, Santa Cruz Biotechnology). Primer used to monitor *Oct4* CR1 in ChIP assays was 5′-CCCACAGCTCTGCTCCTCC-3′ (forward) and 5′-GCTCACCTAGGGACGGTTTC-3′ (reverse). Primer used to monitor *Nanog* CR1 in ChIP assays, was 5′-GGTGGACCCTGCAGGTGGGATTAAC-3′ (forward) and 5′-CCCCTATTCTCCCAGGCACCCAGGC-3′ (reverse).

### Micrococcal nuclease restriction mapping and restriction enzyme accessibility assays

Micrococcal nuclease (MNase) digestion was performed as described (13). Nuclei isolated from ESCs were digested with 5 and 30 U of MNase (Worthington, Lakewood, NJ, USA, www.worthington-biochem.com) at 37°C for 5 min, followed by proteinase K treatment at 37°C overnight. The purified DNA was subjected to Southern blot analysis. Restriction enzyme accessibility assays were carried out as described (13). Isolated nuclei from ESCs were digested with 100 U of SapI, AlwI, FokI, XbaI, PstI, EcoRI or StuI (New England Biolabs, Ipswich, MA, USA, www.neb.com) for 30 min. The purified genomic DNA was re-digested with 100 U of AvaI (for *Nanog* gene) or HindIII (for *Oct4* gene). The digested fragments were analyzed by Southern blot using ^32^P-labeled probe (Figure 5A). All probes were listed in Supplementary Table S1.

### Nucleosome positioning assay

ESCs nuclear extracts were treated with or without 10 U of MNase to digest the nucleosomes. After incubation at 37°C for 30 min, the genomic DNA was purified. The nucleosome positions were determined by the specific amplification of the DNA region with 16 primer sets by qPCR and shown as the ratio of the amount of digested/undigested DNA. Each primer set amplified a ∼80–90-bp product with an average of ∼40-bp overlap to achieve mononucleosome resolution. Data points represent average qPCR signal of DNA protected against MNase digestion from two biological replicates. Primers used in these assays are listed in Supplementary Table S1.

## RESULTS

### RIP140 is required for RA repression of *Oct4* and *Nanog* gene expression

**Figure 1.**
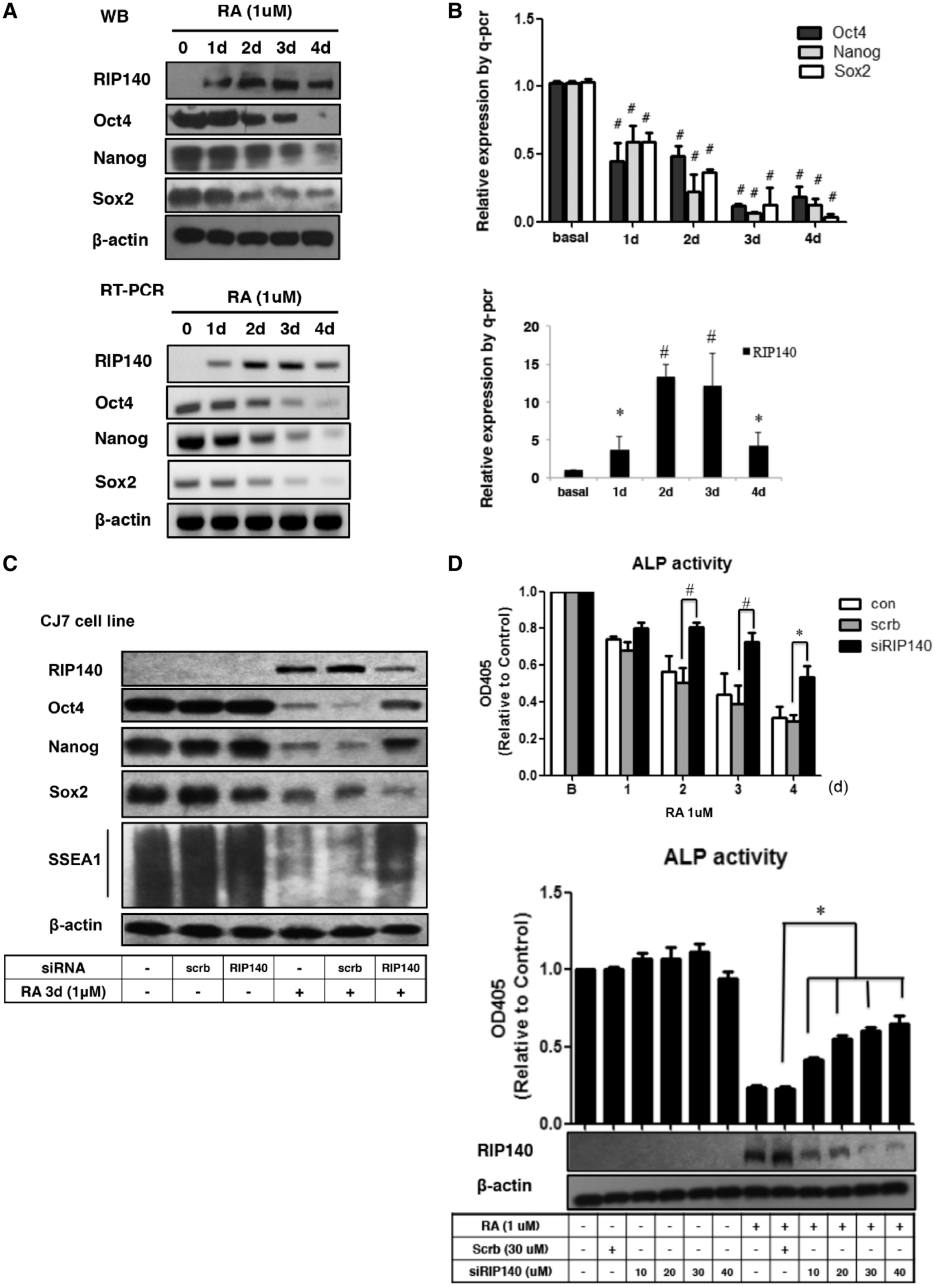
Loss of RA repression of *Nanog* and *Oct4* gene in RIP140-knockdown ESCs. (**A**) Time dependence of Nanog, Oct4, Sox2 and RIP140 protein [detected by western blot (WB)] or mRNA expression (detected by RT-PCR) in ESCs induced with RA (1 µM) for the indicated time intervals. (**B**) qPCR detection of *Oct4*, *Nanog*, *Sox2* and RIP140 mRNA. Data are expressed as mean ± SD of four independent experiments (bar graph). *P < 0.05 and ^#^*P* < 0.01, when compared with the control cells exposed to vehicle alone. (**C**) ES cells were transfected with an RIP140 siRNA (30 nM) for 6 days and sub-cultured on monolayer cultures. Monolayer ESCs were then treated with RA for 3 days. WB analyses were conducted using anti-RIP140, anti-*Nanog*, anti-*Oct4*, anti-*Sox2*, anti-SSEA1 and anti-β-actin Ab as an internal control. (**D**) ESCs were treated with siRNA control (scrb) or RIP140 siRNA (30 nM) for 6 days. Then cells were incubated with 1 µM of RA for the indicated time intervals (upper panel). ESCs were treated with siRNA control (30 nM) or RIP140 siRNA (in the range of 10–40 nM) for 6 days. Cell lysates were examined by WB (RIP140) or ALP activity. Twenty micrograms of cell lysate was incubated with 1× substrate for 60 min and then assayed for ALP activity. Data are expressed as mean ± SEM of three independent experiments (bar graph). *P < 0.05 and ^#^*P* < 0.01, when compared with the cells that were transfected with scrb.

We first determined whether RIP140 was expressed in ESCs and how it reacted to RA treatment. As shown in Figure 1A, RIP140 protein level was increased, whereas Oct4, Nanog and Sox2 protein/RNA levels were decreased, during the course of RA treatment. Real-time quantitative PCR (qPCR) results confirmed this pattern, showing that Oct4, Nanog and Sox2 mRNA levels were reduced, but RIP140 mRNA levels were elevated for the first 3 days of treatment (Figure 1B). This led us to suspect a functional role for RIP140 in ESCs. Using RNA interference (RNAi), we found that RA repression of ESC markers Oct4, Nanog, SSEA1 and alkaline phosphatase (ALP) was effectively rescued by silencing RIP140 (Figure 1C), with the control, β-actin, remained relatively constant. Interestingly, another well-known RA-repressed gene, *Sox2*, was not rescued, suggesting different RA-repressive mechanisms for *Sox2* as compared with *Oct4/Nanog*. We used the same strategy to examine another mouse ESC line B16, and found similar effects as shown in Supplementary Figure S1, which validated that RIP140 played a role in mediating RA-triggered repression of both the *Oct4* and *Nanog* genes, but not the *Sox2* gene. As expected, the ALP activity (Figure 1D) decreased following RA treatment in a time-dependent manner (upper panel), which was partially rescued, dose dependently, by adding siRIP140 (lower panel). These results indicate that RIP140 is involved in RA repression of *Oct4* and *Nanog*, but not the *Sox2* gene, in differentiating ESCs.

### RA-triggered changes in RIP140 and associated chromatin-remodeling components

**Figure 2.**
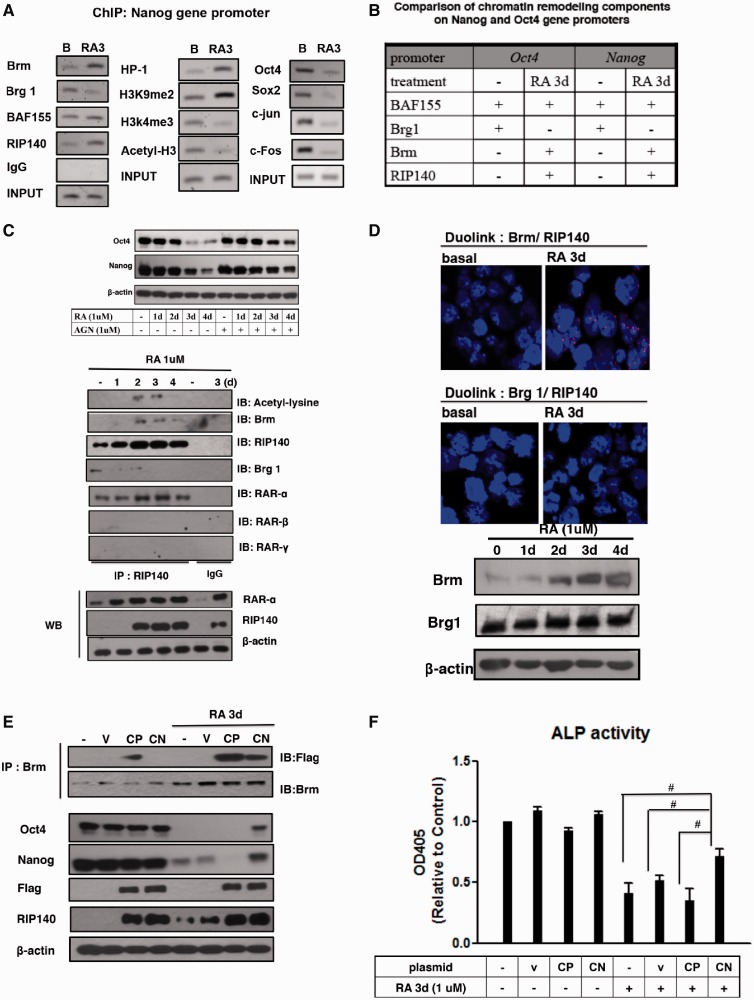
Acetylated RIP140 mediates the recruitment of Brm complex to repress *Nanog* and *Oct4* gene expression in RA-induced differentiation. (**A**) ChIP analyses of chromatin-remodeling factors (Brm, Brg1 and BAF155), histone modification markers (H3k4me3, H3k9me2, H3 acetylation and HP1) and transcription factors (c-Fos, c-Jun, Sox2 and Oct4) on *Nanog* promoter CR1 region. (**B**) A summary of dynamic changes of relevant chromatin-remodeling components compiled from this current study and the previously published data (7). (**C**) Top: the effect of RA is mediated by a canonical pathway that is blocked by a pan-RAR antagonist. Bottom: Co-IP analyses to detect lysine acetylation on RIP140 and the interaction between Brg1, Brm and RAR-α. Lower panels show input controls and IgG as a negative control (**D**) Interaction between RIP140 and Brm, but a lack of interaction between RIP140 and Brg in ESCs. ESCs collected before or after RA stimulation were stained with anti-Brm and -RIP140, or anti-Brg1 and -RIP140, and assessed using the Duolink PLA. Red foci indicate interactions between endogenous Brm and RIP140 proteins. Similar data were observed in ESCs from four different experiments. Brm and Brg1 protein levels after RA treatment were detected by WB. (**E**) Acetyl-RIP140 associates with Brm *in vivo*. Co-IP of acetyl mimetic Flag-RIP140 CP (K158/287Q) and acetyl negative Flag-RIP140 CN (K158/287A) with Brm, and the expression of RIP140, *Oct4*, *Nanog* and Flag proteins were also detected after RA treatment (1 μM, 72 h). CP, Constitutive positive; CN, constitutive negative; IB, immunoblot. (**F**) ESCs were treated with control (empty vector), CN or CP, treated with RA 1 for the indicated time intervals. Twenty micrograms of cell lysate was assayed for ALP activity.

We previously found increased RIP140 recruitment on the *Oct4* gene promoter in RA-treated P19 cells (7), and suspected a similar dynamic change also on the *Nanog* gene locus. As shown in Figure 2A (left) (ChIP), RA triggered RIP140 and Brm association with the *Nanog* gene promoter, in exchange of Brg1 that was initially associated with this chromatin region before RA treatment. BAF155 recruitment to this promoter was not significantly affected by RA treatment.

We assessed several key chromatin marks in this region and found enrichment in the repressive histone mark H3 Lys9 dimethylation and recruitment of HP1, but reduction in the activating histone marks H3 Lys4 trimethylation and H3 acetylation (Figure 2A, middle). This is consistent with the termination in the binding of activating transcription factors (*Oct4*, *Sox2*, *c-Jun* and *c-Fos*) to this chromatin region (Figure 2A, right). In comparison with our previous studies of the *Oct4* gene (7), the dynamic change in the recruitment of RIP140, Brm and Brg1 to the *Nanog* gene in response to RA treatment appeared to be similar (Figure 2B). With this observation, we speculated that RIP140 and Brm might form repressive complexes to coordinate RA repression of *Nanog* and *Oct4* genes, which would more effectively facilitate ESCs’ exit from their pluripotent state in RA-treated cells.

To first determine whether RA repression of these genes occurred along the canonical RAR-mediated pathway, we used a pan-RAR antagonist to block the canonical RAR-mediated pathway. Figure 2C (upper panel) validated that the pan-RAR antagonist, AGN193109 (AGN), blocked this RAR repression process, thus rescuing *Oct4* and *Nanog* expression in RA-treated cells. We then used a Co-IP assay to examine the RIP140-cofactor complex during RA treatment with IgG as a negative control (Figure 2C, middle and lower panel sets). It appears that RIP140 levels are elevated 2–3 days after RA treatment (western blot panel labeled with RIP140) and RIP140 lysine acetylation also peaks on days 2–3 (IB: acetyl-lysine). This is consistent with our previous finding that acetylation on Lys158/Lys287 of RIP140 enhanced its gene repressive activity (20). RA treatment also induced an exchange of the cofactor Brg1 with Brm in the RIP140 complex (IB: Brm and IB: Brg-1). Among the three major RARs (RAR-α, -β and -γ), only RAR-α could be coimmunoprecipitated with RIP140. This result suggests that RAR-α is involved in RA-induced repression of *Nanog* and *Oct4* gene expression in differentiating ESCs, and that RA treatment elevates RIP140 protein level and, correspondingly, increases its lysine acetylation level.

To examine whether endogenous RIP140 directly interacted, or associated, with Brg1 or Brm, we used Duolink assay to detect endogenous protein complexes (complex of two molecules existing in proximity, within a 40-nm range). As shown in Figure 2D (upper panel), RIP140-Brm complexes were evident only in RA-treated cells, but not the untreated ESCs. While we detected a small amount of coprecipitated complex of RIP140 and Brg1 in untreated ESCs (Figure 2C), Duolink assay failed to detect RIP140 and Brg1 complex. This could be due to the fact that RIP140 level was low in untreated ESCs; alternatively, in this complex, RIP140 and Brg1 might not be immediately adjacent to each other. The protein levels of Brg1 and Brm were monitored by western blot (Figure 2D, bottom panels).

Given that lysine acetylation of RIP140 occurred after RA treatment, which correlated with the recruitment of Brm (Figure 2C), we speculated that RIP140 lysine acetylation contributed to its interaction with Brm, thereby downregulating the two RA-target genes. We exploited two previously generated hyper- and hypo-acetylation mutants of RIP140 (RIP140 CP and RIP140 CN, respectively) to ask whether the CN (a dominant negative mutation that was less efficient in recruiting Brm and less repressive) could rescue RA repression of *Oct4* and *Nanog* gene expression, as well as the stem cell property such as their ALP activity, in RA-treated cells. As shown in Figure 2E, RA repression of *Oct4* and *Nanog* was partially rescued by over-expressing CN. As shown in Figure 2F, RA repression of ALP activity was also partially rescued by over-expressing CN mutant. In comparison with the CN mutant, the acetyl-mimetic RIP140 mutant (CP) associated with Brm much strongly, and elicited a much stronger repressive activity than RA treatment alone because the residual *Nanog* expression in RA-treated cells was entirely abolished when CP mutant was introduced into these cells (Figure 2E). Together, these data show that RIP140 and Brm form a repressive complex to mediate RA repression of *Oct4* and *Nanog* genes and promote ESCs’ exit from their ground state. Further, lysine acetylation of RIP140 potentiates this activity.

### RA-triggered alterations in RIP140/chromatin-remodeling machineries on *Oct4* and *Nanog* gene promoters

**Figure 3.**
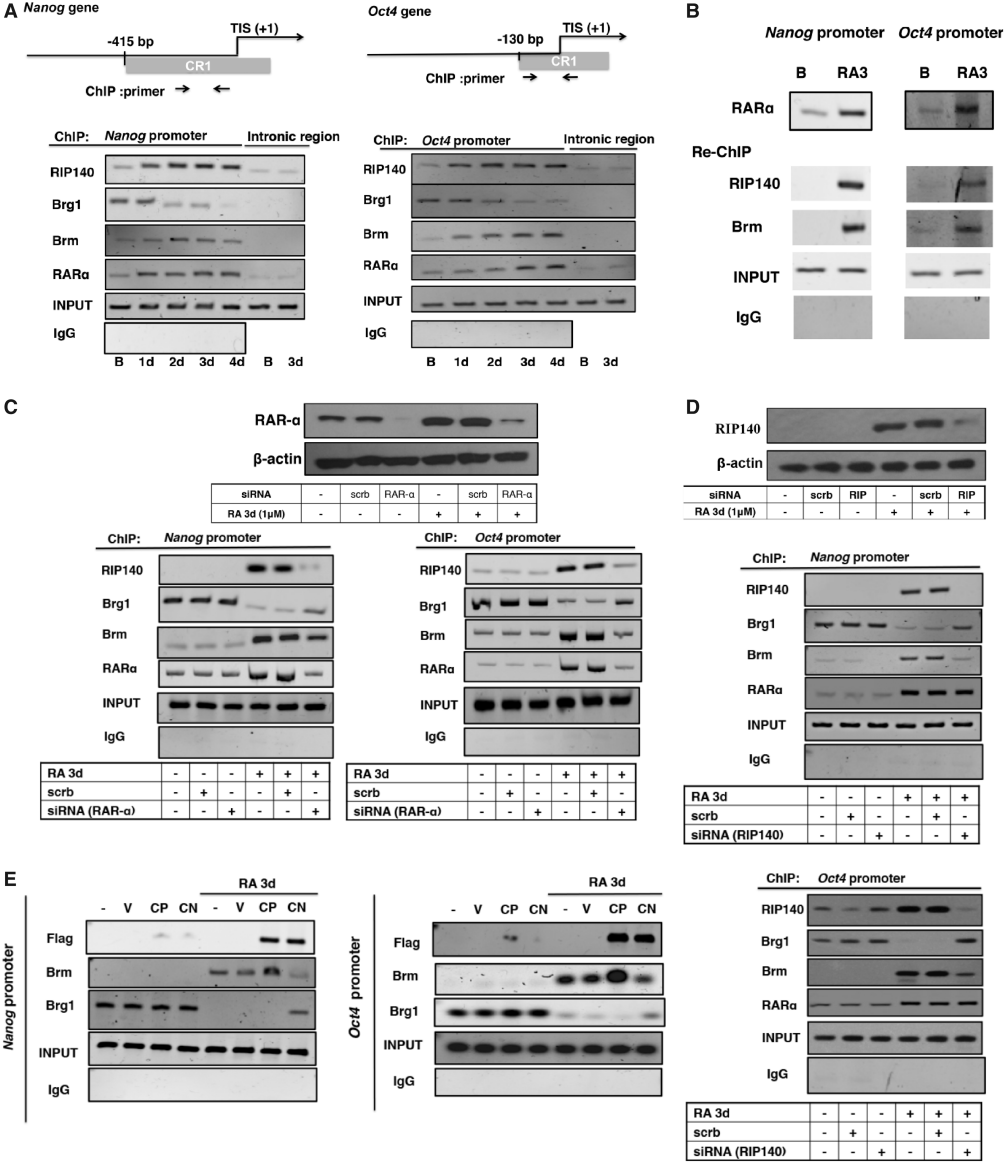
RAR-α is involved in RIP140/Brm repressive complex in RA-induced ESC differentiation. (**A**) ChIP assay examining the kinetics of RIP140, Brm, Brg1 and RAR-α recruitment on the promoters of *Nanog* or *Oct4* genes. Primers specific to the introns of *Nanog* and *Oct4* were used for negative controls. The positions of primer pairs are depicted on the map. (**B**) Re-ChIP to monitor endogenous RAR-α associated with RIP140 and Brm on *Nanog* or *Oct4* gene promoter on day 3 of RA treatment. (**C**) Upper: WB showing the expression of RAR-α in ESCs transfected with scrb siRNA or RAR-α siRNA. Lower: ESCs were transfected with scrb or siRNA specific to RAR-α, and then were treated with RA for 72 h after 6 days of siRNA transfection. ChIP assay was conducted to examine the recruitment of Brm, Brg1, RAR-α and RIP140 on the promoters. (**D**) Upper: WB showing the expression of RIP140 in ESCs transfected with scrb siRNA or RIP140 siRNA. Lower: RIP140 is required for Brm/Brg1 exchange. ESCs were transfected with scrb or siRNA specific to RIP140, treated with RA for 72 h after 6 days of siRNA transfection. ChIP assay was conducted to examine the recruitment of Brm, Brg1, RAR-α and RIP140 on the promoters. (**E**) RIP140 lysine acetylation is important for Brm/Brg1 exchange. ESCs were treated with control (empty vector), CN or CP, then treated with RA for 72 h. ChIP assay was conducted to examine the Brm, Brg1 and RIP140 recruitment to the promoters of the *Nanog* or *Oct4* genes.

To decipher the mechanism of action of RAR-α, RIP140 and Brm, ChIP was performed to first monitor the dynamic changes in the recruitment of these repressive components on the *Nanog* and *Oct4* promoters (Figure 3A). RA induced increasing binding of RAR-α, RIP140 and Brm on *Nanog* and *Oct4* promoters, which gradually displaced Brg1. In consistence with previous results, we did not detect RIP140/Brm association with the *Sox2* promoter (Supplementary Figure S2), supporting differences in RA repression of *Oct4* and *Nanog* as compared with *Sox2*. To determine the repressive RIP140 complex on *Oct4* and *Nanog* promoters, we used repeated ChIP (Re-ChIP) to monitor the endogenous components (RIP140 and Brm) associated with RAR-α on these chromatin targets. The first ChIP (Figure 3B, top) showed RAR-α recruitment after RA treatment. Re-ChIP revealed that RIP140 and Brm co-occupancy with RAR-α on these promoters after RA treatment, indicating that RAR-α, RIP140 and Brm could form repressive complex on the promoters of *Nanog* and *Oct4* after RA treatment.

The essential functional roles for RAR-α and RIP140 in RA-induced recruitment of Brm to displace Brg1 on these promoters were validated using siRNA knockdown followed by ChIP experiments. RAR-α silencing resulted in a marked decrease in RIP140 and Brm recruitment, but increased Brg1 recruitment, after RA treatment (Figure 3C). The efficiency of RAR-α silencing was confirmed (shown in the upper panel). These results suggested that RA-induced recruitment of RIP140 and Brm to these promoters depended on RAR-α. Figure 3D showed the effects of silencing RIP140. The efficiency of RIP140 silencing was confirmed (upper panel). Depletion of RIP140 apparently blocked RA-induced recruitment of Brm on both *Oct4* and *Nanog* promoters, and rescued, at least partially, the displacement of Brg1 from these two promoters.

We further confirmed that RIP140’s lysine acetylation is important for recruiting Brm. As shown in Figure 3E, introducing the acetyl-mimetic RIP140 into RA-treated cells rendered more effective recruitment of Brm to these two promoters, whereas introducing the hypoacetylation mimetic RIP140 not only reduced Brm recruitment but, in fact, rendered Brg1 staying on both promoters. All together, the data show that RAR-α is essential for RIP140-Brm complex recruitment and RIP140 is important for Brm recruitment to displace Brg1. Further, lysine acetylation of RIP140 seems to be one key factor modulating the efficiency of the exchange of Brg1 with Brm.

### Brm in RA-induced nucleosome insertion on *Nanog* promoter

**Figure 4.**
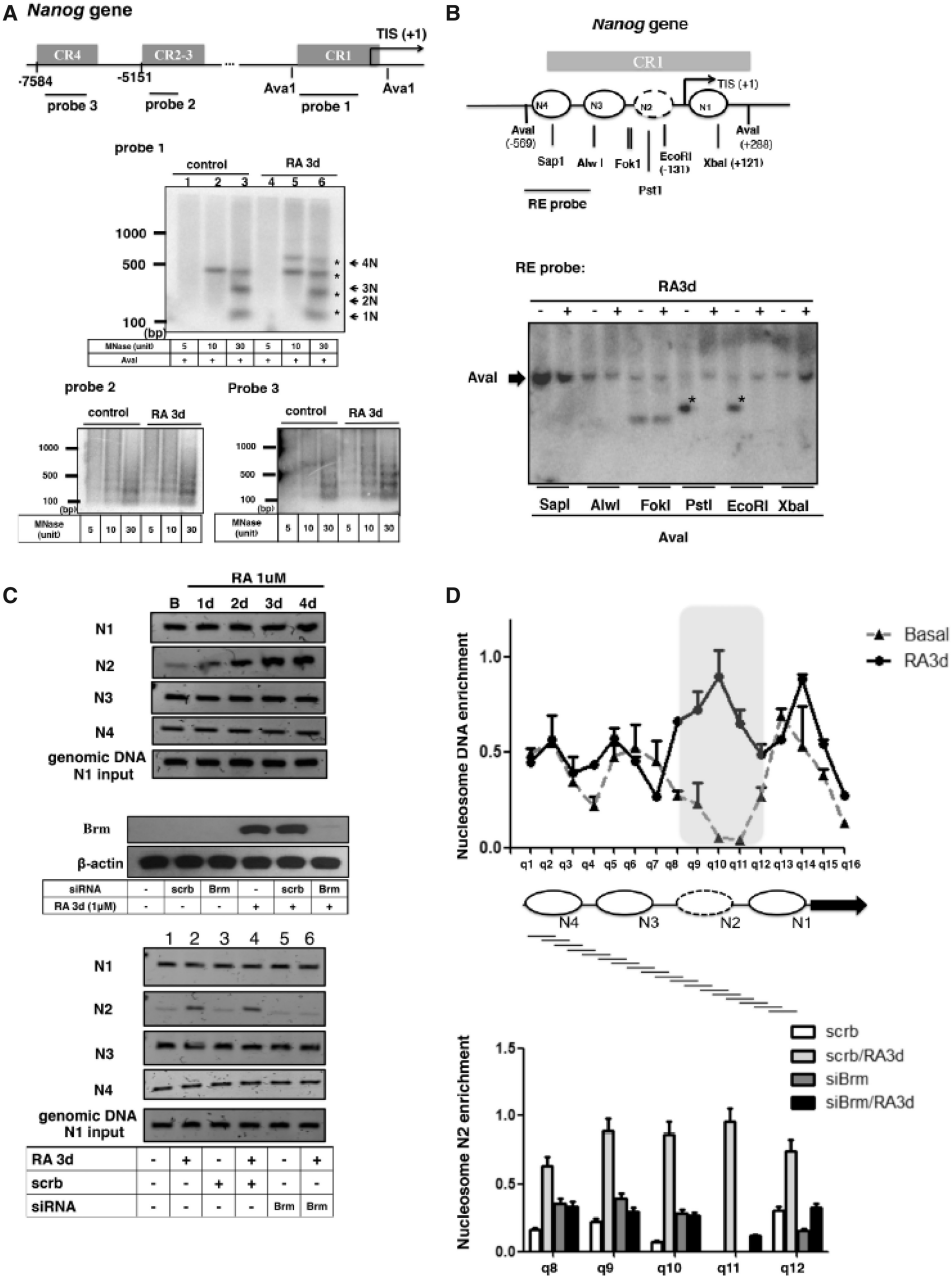
Chromatin-remodeling (nucleosome insertion and rearrangement) on *Nanog* gene promoter. (**A**) ESCs were treated with RA for 3 days, then treated with 5, 10 and 30 U of MNase for 6 min at 37°C. Extracted chromatin DNA was separated on 1.5% agarose gels followed by Southern blot hybridization with ^32^P-labeled 500-bp probes specific to three regions on the *Nanog* promoter. Positions of probes are depicted on the map. (**B**) Restriction enzyme accessibility of *Nanog* promoter region in ESCs. Asterisk marks the diagnostic band indicative of each sensitive site. The results are summarized and shown on the map above these blots. Restriction sites are labeled under the map. (**C**) N1, N2, N3 and N4 PCR fragments, amplified from mononucleosomal DNA using the primers a/b (N1), c/d (N2), e/f (N3) and g/h (N4). Primer sequences were listed in Supplementary Table S1. Genomic DNAs isolated from 1 × 10^6^ cells were used for N1 amplification as control (upper panel). ESCs were transfected with siRNA specific for Brm or siRNA control. Changes in mononucleosome formation on the *Nanog* gene promoter were monitored (bottom panel). (**D**) Upper: nucleosome occupancy on the *Nanog* promoter in ESCs with or without RA treatment. The gray-highlighted region (q8–12) represents the location of the N2 nucleosome formation. Lower: analysis of nucleosome occupancy at the q8–12 regions of *Nanog* in control and Brm-knockdown ES cell by the MNase resistance assay. Data points represent average qPCR signals from two independent experiments.

We previously reported RA-induced chromatin-remodeling process of the *Oct4* proximal promoter, which involved the insertion of a nucleosome (7,24). For comparison, here we first determined the basal chromatin conformation of the *Nanog* gene promoter in stem cells, and examined the mode of remodeling that might be triggered by RA. We used MNase mapping to determine four principal regulatory regions of the *Nanog* gene promoter, the conserved regions (CR) 1, CR2-3 and CR4 (map shown on the top of Figure 4A). As shown in Figure 4A, clear regular nucleosomal arrays appeared on all these regulatory regions in both undifferentiated stem cells (control) and RA-treated cells. Notably, in RA-treated cells, an additional nucleosome was detected using probe 1 (middle panel), but not probes 2 or 3 (lower panel), suggesting lengthening, or an insertion of one nucleosome in the CR1 region of RA-treated cells (comparing lane 7 with lane 3 of probe 1 data, nucleosomes marked with asterisks). To validate this change, we conducted restriction accessibility assay to focus on CR1 region as shown in Figure 4B. RA treatment clearly altered the restriction accessibility on both PstI and EcoRI sites (marked with asterisk) but not on SapI, AlwI, FokI or XbaI sites, suggesting nucleosome repositioning on the region spanning PstI site and EcoRI site after RA treatment. According to the MNase data (Figure 4A), we predicted that CR1 initially assembled three nucleosomes in stem cells and then inserted one additional nucleosome in RA-treated cells. We then used a PCR-based method to assess locations of each nucleosome within the CR1 region (25) in stem cells versus RA-treated cells (Figure 4C). This method uses PCR primer pairs to amplify specific chromatin segments spanning selected regions that are predicted to undergo remodeling. The rational is, if this segment is occupied by a nucleosome, it will be amplified even after MNase digestion. On the contrary, if this segment is not occupied by nucleosomes, it will not be amplified after MNase digestion. Based on restriction data (Figure 4B), we predicted four nucleosomes formed on CR1 (N1 fragment spans Xba1 site, N2 fragment spans EcoRl and Pstl sites; N3 fragment spans Alwl site; N4 fragment spans Sapl site). As shown in the upper panel of Figure 4C, N2 was weakly detected in stem cells and increasingly detected in RA-treated cells (lane N2), supporting the addition/insertion of N2 in RA-treated cells. For an internal control, genomic DNA was always included in the assay to confirm equal inputs in these assays. We suspected that Brm played a role in the insertion of N2. As shown in bottom panel of Figure 4C, silencing Brm (efficiency validated in the middle panel) blocked RA-induced formation of N2 nucleosome (Figure 4C lower panel). We further conducted a nucleosome scanning experiment by designing 16 overlapping qPCR primers (q1–q16) that each amplified an 80–90 bp product (amplicon). Each amplicon created had an average of a 40-bp overlapped sequence to achieve mono-nucleosome resolution (Figure 4D). Each data point represents the average qPCR signal of DNA protected against MNase digestion (nucleosomal region is protected against MNase digestion). In stem cells (Basal), three discrete peaks appeared, supporting three nucleosomes within the CR-1 region; whereas in RA-treated cells, four peaks appeared (the new peak detected mainly in the N2 position), and a shift in the positions of the original three nucleosomes was detected, supporting four nucleosomes formed in CR-1, which originally contained three nucleosomes. We then focused on N2 formation (regions q8–q12). Silencing Brm in RA-treated cells dramatically reduced the efficiency of N2 formation (Figure 4D bottom panel). These experiments show that three nucleosomes are readily present in the CR1 region of the *Nanog* gene in undifferentiated stem cells, which then forms four nucleosomes on RA induction. Brm is important for the insertion of N2 into the CR1 region of the *Nanog* gene in RA-treated cells.

Combining data shown in Figure 4C and D, we suggest that there is a new nucleosome inserted, or slid, into this region following RA treatment, which condenses this chromatin segment.

### Brm and RIP140 are required for RA-induced chromatin-remodeling of *Oct4* and *Nanog* genes

**Figure 5.**
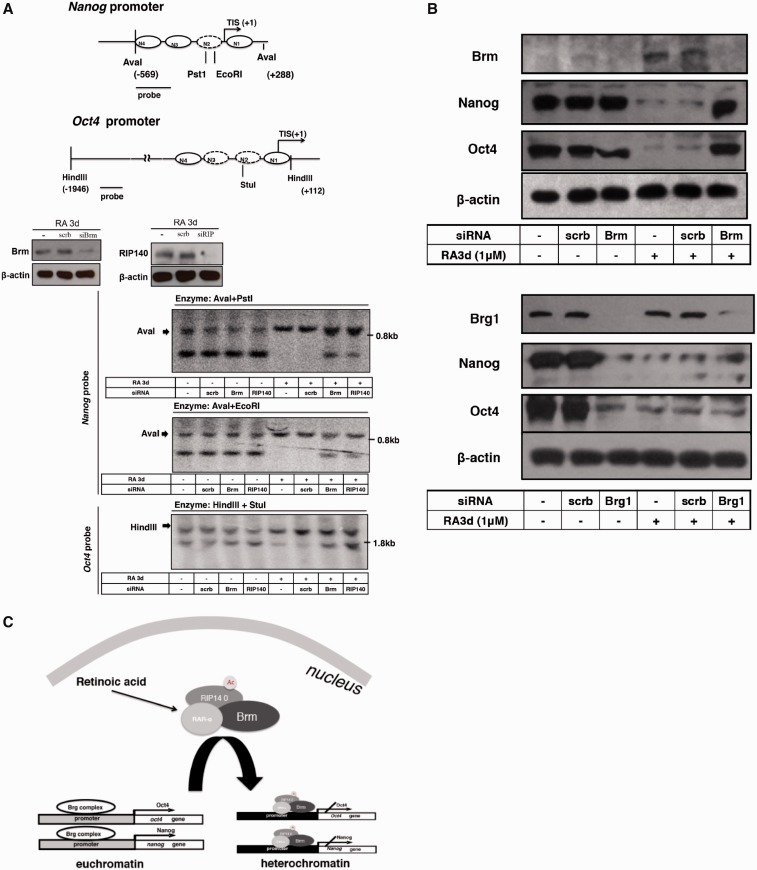
RIP140/Brm complex is required for RA-induced remodeling of *Nanog* and *Oct4* promoters. (**A**) ESCs were transfected with RIP140 or Brm siRNA and cultured without (–) or with 1 μM RA for 3 days. The efficacy of silencing is shown in the middle panel. Nuclei were digested with PstI, EcoRI or Stul at 37°C for 30 min. Purified genomic DNA (20 μg) was digested with Aval or Hindlll and separated on a 1% agarose gel. Southern blot was performed with a ^32^P-labeled 300- or 200-bp fragment specific to *Oct4* or *Nanog* promoter fragment. Restriction maps of *Oct4* and *Nanog* promoters are shown on the top (**B**) ESCs were transfected with scrb or siRNA specific for Brm, Brg1, and then cells were treated with RA for 72 h after 6 days of siRNA transfection. Levels of Brm, Brg1, *Nanog*, *Oct4* and β-actin were determined on WB. (**C**) A model of RAR-α/RIP140/Brm complex formation and acting on *Oct4* and *Nanog* genes after ESCs differentiation.

Finally, given that *Nanog* seemed to undergo a similar nucleosome insertion process as that of *Oct4* gene (7), we suspected a role for RIP140 and Brm in coordinated remodeling of *Oct4* and *Nanog* genes following RA treatment, particularly on the N2 position. We used restriction accessibility assays to specifically monitor the accessibility of N2 in both the *Nanog* and *Oct4* promoters in the absence and presence of RIP140 or Brm. These regions are depicted on the upper two maps—PstI and EcoRI both can assess the accessibility of N2 on the *Nanog* promoter; whereas StuI can assess N2 on the *Oct4* promoter. As shown in lower three panels of Figure 5A, silencing RIP140 or Brm (as compared with no siRNA/RA3d or scrb/RA3d controls) reduced RA-triggered N2 formation on *Nanog* (decreased accessibility to AvaI+PstI and AvaI+EcoRI) and *Oct4* (decreased accessibility to HindIII+StuI) promoters. The efficiency of Brm and RIP140 silencing was confirmed (middle panel). Finally, we examined whether silencing Brm would blunt RA repression of *Oct4* and *Nanog* genes. As shown in Figure 5B upper panel, silencing Brm rescued the expression of both *Oct4* and *Nanog* that would have been repressed by RA treatment. As predicted, silencing Brg1 resulted in a loss of expression of both *Oct4* and *Nanog* (Figure 5B, bottom panel).

## DISCUSSION

In this study, we propose an RA-induced epigenetic mechanism for the exit of ESCs from their ground-state pluripotency to lineage commitment. Our results demonstrate that the RAR-α/RIP140/Brm complex is a critical component of the machinery that mediates RA repression of two key stemness genes, *Nanog* and *Oct4,* through chromatin-remodeling. The remodeling processes of *Nanog* and *Oct4* gene promoters both require a switch of Brg1 and Brm, which is triggered by RIP140 lysine acetylation. Figure 5C depicts the RA-induced coordinated RAR-α/RIP140/Brm complex formation on *Oct4* and *Nanog* promoters.

RA exerts pleiotropic effects in ESC differentiation and mammalian development. For ESCs, RA inhibits their self-renewal and promotes their neurogenesis, which is mediated by the action of RARs (26), and for most genes, RA is thought to activate target gene expression. However, how RA acts on key stemness factors for ESCs to exit from their ground states and how RA induces repressive chromatin-remodeling remain unclear. Our data suggest that RA repression of *Oct4* and *Nanog* is an RAR-mediated direct effect on the chromatin, as treating cells with an RAR pan-antagonist (AGN) rescued *Nanog* and *Oct4* gene expression (Figure 2C), and silencing RAR-α in RA-treated cells (Figure 3C) could rescue RA-triggered Brg1/Brm exchange. Furthermore, RIP140/Brm complex is recruited by RAR-α on both *Nanog* and *Oct4* gene promoters to engage repressive chromatin-remodeling by inserting one nucleosome into a specific region of their proximal promoters, CR1. RIP140 is known as a corepressor for most nuclear receptors. This study extends our knowledge and establishes that RIP140 can also help recruiting chromatin-remodeling component Brm, in the case of *Oct4* and *Nanog,* for gene repression. Most interestingly, the recruitment of Brm by RIP140 requires its specific lysine acetylation, which has been previously shown to enhance RIP140’s gene repressive activity (21). Another important finding in this study has to do with the remarkably similar RA-triggered repressive remodeling processes of both *Oct4* and *Nanog* genes, that both occur mainly within the proximal promoter region CR-1 and both involve the addition/insertion of new nucleosomes, presumably to make this region more compact. This requires both RIP140 and Brm, and appears to be entirely different from RA repression of another important RA-target gene, *Sox2*. The detailed mechanism underlying the seemingly coordinated remodeling processes of *Oct4* and *Nanog* genes in ESCs awaits further studies.

Both Brm- and Brg1-centered SWI/SNF complexes mediate either transcriptional activation or repression, depending on the interacting subunits or transcription factors (27–29). For example, the mSin3A/HDAC corepressor complex has been found in Brm or Brg1 complexes (28,30) as well as the HAT-P300 coactivator complex (31,32). Brg1 and Brm have different expression profiles. Brg1 protein level is maintained relatively constant in all cell types, whereas Brm level is low or absent in stem cells, and is only induced during differentiation (33), which is consistent with our data (Figure 2D, bottom panel). Thus, the main determinant of the type of chromatin-remodeling that could occur in ESCs seems to reside on the level of Brm.

Similar to previous findings (8,9), Brg1 associates with *Nanog* and *Oct4* gene promoters (Figure 3A) to maintain these genes’ activity in stem cells (Figure 5B, bottom panel), indicating that Brg1 may be involved in regulating stem cell pluripotency. Brg1 is then completely replaced by Brm, via the action of RIP140, on the promoters of *Nanog* and *Oct4* after RA treatment (Figure 3A and D). Brm and RIP140 together then occupy the promoters of *Nanog* and *Oct4* genes and contribute to their chromatin compaction after RA treatment (Figures 3A, 4C and 5A). These results strongly suggest that the Brg1/Brm switch, modulated by lysine acetylation of RIP140, may represent an ‘on–off switch’ for the expression of these key stemness genes. Notably, knocking down RIP140 prohibits Brg1 from disassociating from gene promoter, which seems more effective for the *Oct4* than the *Nanog* gene (Figure 3D). How this difference may happen remains to be examined. Further, Brg1/Brm switch has also been observed on IFNγ-activated sequences (GAS) under heat shock and/or IFNγ treatment (29). It will be interesting to examine whether RIP140 may also play a role in Brg1/Brm switch on GAS.

RIP140 can also directly interact with other repressive cofactors such as CtBP (34) and HDAC3 (35), suggesting that RIP140 could be a nexus for multiple histone modification and/or chromatin-remodeling factors for gene silencing. How RIP140 dynamically recruits these various factors is still unclear; presumably it depends on the expression levels of these factors. However, protein modification status of RIP140 is also crucial, as demonstrated in this study that lysine acetylation appears to be critical for recruiting Brm. How protein modification of RIP140 may modulate the composition of various RIP140 complexes will be an important topic in future studies.

This study reports, for the first time, that RAR-α, RIP140 (particularly its lysine-acetylated form) and Brm are required for coordinated chromatin-remodeling of *Oct4* and *Nanog* genes in RA-treated ESCs. Interestingly, the major remodeling process occurs in the CR1 region of both genes, and involves the insertion of new nucleosomes. This may contribute to the coordinated regulation of these two stemness genes. Whether this requires any spatial coordination of the two gene loci remains to be investigated.

## SUPPLEMENTARY DATA

Supplementary Data are available at NAR Online.

## FUNDING

NIH [DK54733, DK60521], the Dean’s Commitment and the Distinguished McKnight Professorship of University of Minnesota (to L.N.W.). Funding for open access charge: NIH [DK54733 and DK60521].

*Conflict of interest statement*. None declared.

## Supplementary Material

Supplementary Data
