# Supplementary material for: Coordinated repressive chromatin-remodeling of Oct4 and Nanog genes in RA-induced differentiation of embryonic stem cells involves RIP140
Source: Nucleic Acids Res. 2014 Jan 30;42(7):4306–17. doi: 10.1093/nar/gku092 (PMC3985664; doi:10.1093/nar/gku092)
Supplement: Supplementary Data [file supp_42_7_4306__index.html]

Coordinated repressive chromatin-remodeling of Oct4 and Nanog genes in RA-induced differentiation of embryonic stem cells involves RIP140 — Coordinated repressive chromatin-remodeling of Oct4 and Nanog genes in RA-induced differentiation of embryonic stem cells involves RIP140 — Supplementary Data 

# Coordinated repressive chromatin-remodeling of *Oct4* and *Nanog* genes in RA-induced differentiation of embryonic stem cells involves RIP140

## Supplementary Data

files

**Files in this Data Supplement:**

- Supplementary Data - doc file
