# Supplementary material for: Coordinated repressive chromatin-remodeling of Oct4 and Nanog genes in RA-induced differentiation of embryonic stem cells involves RIP140
Source: Nucleic Acids Res. 2014 Jan 30;42(7):4306–17. doi: 10.1093/nar/gku092 (PMC3985664; doi:10.1093/nar/gku092)
Supplement: Supplementary Data [file supp_gku092_nar-03384-v-2013-File002.doc]

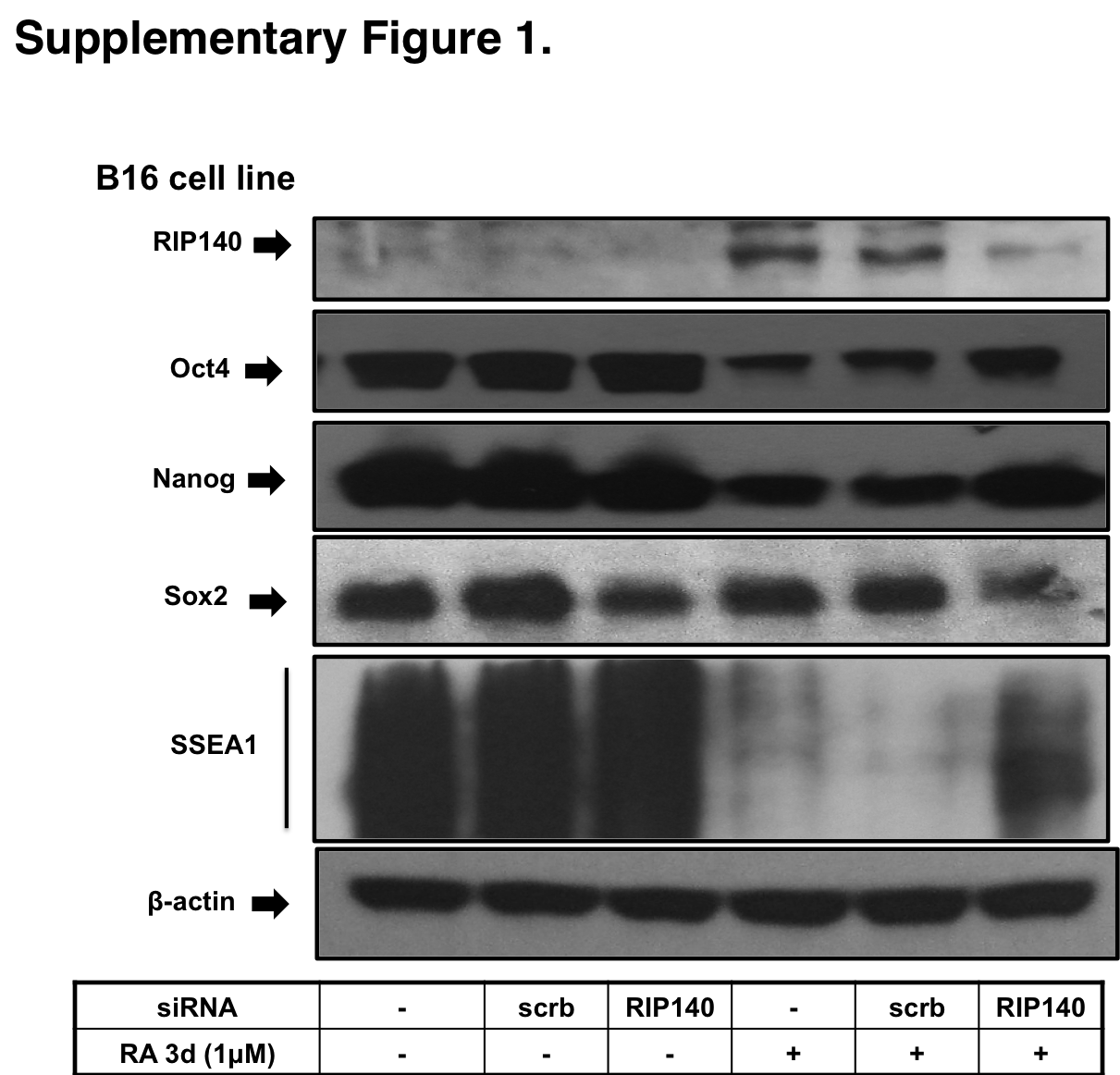


Supplementary Fig. 1.

ES cell were transfected with an RIP140 siRNA (30 nM) for 6 days and sub-cultured on monolayer cultures. Monolayer ESCs were then treated with RA for 3 days. Western blot analyses were conducted using anti-RIP140, anti-Nanog, anti-Oct4, anti-Sox2, anti-SSEA1 and anti-β-actin Ab as an internal control.


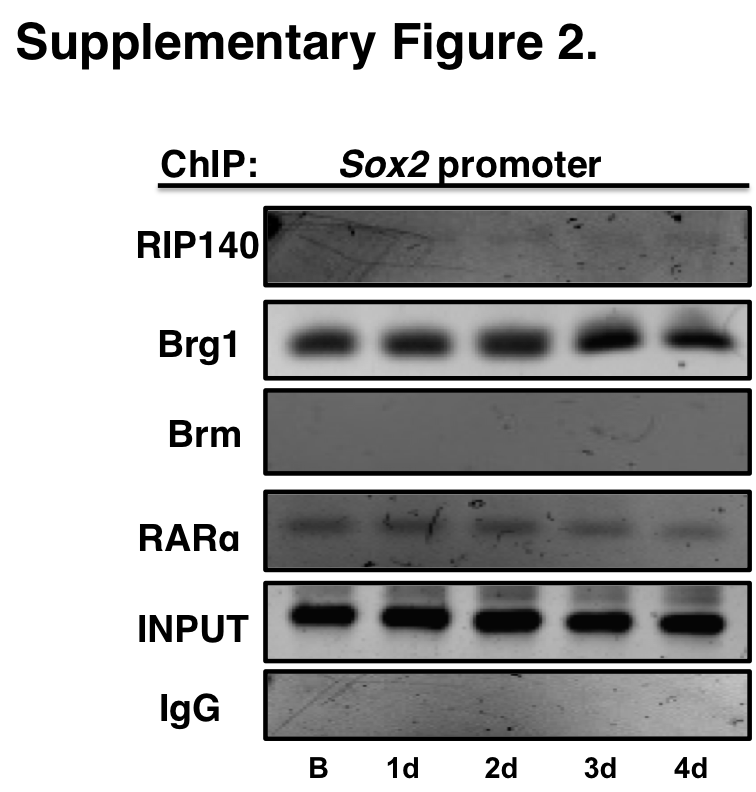


Supplementary Figure 2.

ChIP assay examining the kinetics of RIP140, Brm, Brg and RAR-α recruitment on the promoters of *Sox2* gene.

Supplementary Table 1

PCR

| Primers | Sequence (5'-3') |
| --- | --- |

a (N1) 5^0^-AGGCTGCGGCTCACTTCCTTCTGAC-3^0^

b (N1) 5^0^-AGAAGGAAAGATCATAGAAAGAAGA-3^0^

c (N2) 5^0^-GGTGGACCCTGCAGGTGGGATTAAC-3^0^

d (N2) 5^0^-CCCCTATTCTCCCAGGCACCCAGGC-3^0^

e (N3) 5^0^-CAGATCCCCCACTTGACCTGAAACT-3^0^

f (N3) 5^0^-TTATTCATCTTTTAACCACGGCTGC-3^0^

g (N4) 5^0^-GCACTTTTGGAGGGAAGATTTCCCC-3^0^

h (N4) 5^0^-CACGGCTTTCCTTTCAGCACTCAGC-3^0^

Nanog intron (F) 5^0^-GGTATGGATCCCAGATTTCC-3^0^

Nanog intron (R) 5^0^- GGACATAGATGGATCAGGAT-3^0^

Oct4 intron (F) 5^0^- GTGAGGAGTGGCAGGATGTGTG-3^0^

Oct4 intron (F) 5^0^-TGCGAGCTCCGGTTGGAGAG-3^0^

Micrococcal Nuclease Nucleosome Mapping

| Primers | Sequence (5'-3') |
| --- | --- |

MNase CR1 (F) 50- AGTTCAGGGTAGGCTCTCTG-30

MNase CR1 (R) 50- AATGGCCTTGGCTGGACAAT-30

MNase CR2-3 (F) 50- GGCAGACGGGTCTCCAGTAG -30

MNase CR2-3 (R) 50-GTAAGAGCTGGGAGGAGTCCAGA-30

MNase CR4 (F) 50- CCAAGCCTGGCTTAAGATTC-30

MNase CR4 (R) 50- AGCCACCTTGTAGTTTGTTG-30

Restriction Enzyme Accessibility Assays

| Primers | Sequence (5'-3') |
| --- | --- |

RE Nanog (F) 5^0^ –GGGAAGTTTCAGGTCAAGTG-3^0^

RE Nanog (R) 5^0^-TAAGCAGGACACAGGCTCTT-3^0^

RE Oct4 (F) 5^0^-AGTTCAGGGTAGGCTCTCTG-3^0^

RE Oct4 (R) 5^0^- AATGGCCTTGGCTGGACAATC-3^0^

Nucleosome positioning assay

| Primers | Sequence (5'-3') |
| --- | --- |

NP-1 (F) 5^0^-GAGTCTTAAGCAGGACACAGGCTCT-3^0^

NP-1 (R) 5^0^-AACCTGGGGAAATCTTCCCTCCAAA-3^0^

NP-2 (F) 5^0^-GCACTTTTGGAGGGAAGATTTCCCC-3^0^

NP-2 (R) 5^0^-CACGGCTTTCCTTTCAGCACTCAGC-3^0^

NP-3 (F) 5^0^-AAGAAACGCTGAGTGCTGAAAGGAA-3^0^

NP-3 (R) 5^0^-CTACAGACATGAAAGAGTCAGACCT-3^0^

NP-4 (F) 5^0^-CAGCAAGGTCTGACTCTTTCATGTC-3^0^

NP-4 (R) 5^0^-AGTGGGAAGTTTCAGGTCAAGTGGG-3^0^

NP-5 (F) 5^0^-CAGATCCCCCACTTGACCTGAAACT-3^0^

NP-5 (R) 5^0^-TTATTCATCTTTTAACCACGGCTGC-3^0^

NP-6 (F) 5^0^-GAGGTGCAGCCGTGGTTAAAAGATG-3^0^

NP-6 (R) 5^0^-GGGGGCATCCTCTGATCTAAAGACA-3^0^

NP-7 (F) 5^0^-GGGGGGGGATGTCTTTAGATCAGAG-3^0^

NP-7 (R) 5^0^-CAAAAGAAGCTGTAAGGTGACCCAG-3^0^

NP-8 (F) 5^0^-CCCTCCCAGTCTGGGTCACCTTACA-3^0^

NP-8 (R) 5^0^-TCACAGTTAATCCCACCTGCAGGGT-3^0^

NP-9 (F) 5^0^-GGTGGACCCTGCAGGTGGGATTAAC-3^0^

NP-9 (R) 5^0^-CCCCTATTCTCCCAGGCACCCAGGC-3^0^

NP-10 (F) 5^0^-GTAAAGGGTAGGTCCTGCTCCTCCCC-3^0^

NP-10 (R) 5^0^-GCGTGGGTGCCGCCTGGGTGCCTG-3^0^

NP-11 (F) 5^0^-GAGGCTTGAGGGGGGAGGAGCAGGA-3^0^

NP-11 (R) 5^0^-CCAAATCAGCCTATCTGAAGGCCAA-3^0^

NP-12 (F) 5^0^-TGAGCCGTTGGCCTTCAGATAGGCT-3^0^

NP-12 (R) 5^0^-GTCAGAAGGAAGTGAGCCGCAGCCT-3^0^

NP-13 (F) 5^0^-AGGCTGCGGCTCACTTCCTTCTGAC-3^0^

NP-13 (R) 5^0^-AGAAGGAAAGATCATAGAAAGAAGA-3^0^

NP-14 (F) 5^0^-CTCTTCTTTCTATGATCTTTCCTTC-3^0^

NP-14 (R) 5^0^-GAGGGAAGGGATTTCTGAAAAGGTT-3^0^

NP-15 (F) 5^0^-GTTGTTGCCTAAAACCTTTTCAGAA-3^0^

NP-15 (R) 5^0^-GCAAACTGTGGGGACCAGGAAGACC-3^0^

NP-16 (F) 5^0^-CTGACATGAGTGTGGGTCTTCCTGG-3^0^

NP-16 (R) 5^0^-TGATGAGGCGTTCCCAGAATTCGAT-3^0^
